# The projective wave theory of consciousness

**DOI:** 10.3389/fpsyg.2026.1674983

**Published:** 2026-02-25

**Authors:** Robert Worden

**Affiliations:** Active Inference Institute, Crescent City, CA, United States

**Keywords:** Bayesian balance, computational functionalism, consciousness, encoding and decoding of information, Fourier transform, thalamus, wave excitation, projective transform

## Abstract

Many theories of consciousness propose that consciousness arises from neural computation in the brain. All information in a neural computer is physically encoded, but consciousness contains un-encoded information about local space. The information required for decoding does not reside in the brain. So consciousness cannot arise from encoded neural information; but it could arise from un-encoded information, such as an analogue model of local 3-D space. This paper proposes that the mammalian brain holds an analogue model of 3-D space, as a wave excitation in the thalamus. The wave stores information in a Fourier transform of space, like a hologram. Neurons couple to the wave, and the wave is the source of consciousness. Such a wave has not been detected in the brain; but there are reasons why it has not yet been detected, and there are reasons for a wave to have evolved. There is indirect evidence for a wave in the mammalian thalamus, and in the central body of the insect brain. This paper is an initial conceptual outline of a projective wave theory of consciousness, in which phenomenal consciousness arises solely from a wave excitation in the thalamus. Neuronal activity maintains the wave, but has no direct link to consciousness. Such a theory is capable of agreeing well with the spatial form of our conscious experience. It avoids the decoding problem of neural theories of consciousness, and has the potential for a positive Bayesian balance between the complexity of its assumptions and the data it fits; this is reason to investigate it further. The theory would explain why consciousness evolved, and be falsifiable. Many details of the theory remain to be worked out.

## 1 Introduction

There is a serious difficulty for many current computational theories of consciousness (Worden, 2025). Phenomenal consciousness contains information about external things. Where does that information come from? The usual answer is that the brain is a neural computer, and the information in consciousness comes from neural computation processes in the brain.

That answer does not work. The reason is that all information in a computer is physically encoded (in a brain, dynamic information is encoded as neural spike trains). Without decoding, that information is not information about any defined thing. Neural firing is like an encrypted message. It would need to be decoded (decrypted) to give the information in consciousness. The information required to decode it includes information such as: which of the 0.86 * 10^11^ neurons in the brain (Avezedo, 2009) encode information about external things? how is that information encoded—as neural firing rates, correlations, synchrony, or in other ways? The decoding information does not exist inside the brain. Without it, there is an unlimited number of possible decodings, most of them meaningless. Hence, the physical events in a brain do not contain the information we experience in consciousness.

This follows from an analysis of the nature of computation—which implies that computation is not just a physical process, but is an interpretation (or decoding) of a physical process (Horsman and Stepney, 2014; Stepney and Kendon, 2021).

For computational theories of consciousness, this is not just a difficulty; it is an insurmountable barrier. It implies that the thesis of computational functionalism (Putnam, 1988; Chalmers, 2012; Butlin and Long, 2023) is incorrect. Therefore many theories of consciousness that depend on it, such as Brown et al. (2019); Deane (2021); Dehaene and Changeux (2011); Lamme (2020); Tononi (2012), are incorrect. To account for consciousness, there must be something else, as well as neural computation, happening in brains.

In Worden (2025), I suggested what that something else could be. As well as a neural computer, the brain may contain an analogue model of local 3-D space. There is information in an analogue model of reality which requires no decoding. Hence such an analogue model could be the source of phenomenal consciousness.

This paper develops that proposal, showing that it can give a testable theory of phenomenal consciousness (Block, 1978). This is the Projective Wave theory of consciousness. The theory is simple, it fits with what we know about the brain, and has benefits over neural theories of consciousness. It agrees well with evidence about the spatial properties of consciousness, and it describes how consciousness has evolved to support a vital function in brains. It could be falsified (Sprenger and Hartmann, 2019), if no wave excitation is found in the brain. Scientific theories should be falsifiable.

Section 2 of the paper recapitulates the arguments in Worden (2025) that purely computational models of the brain cannot account for phenomenal consciousness, and that something else is required in the brain.

Section 3 suggests that the ‘something else’ in the brain could be an analogue model of local space—a model which (unlike neural representations of space) is not heavily encoded—and so could be the basis of consciousness. This proposal raises questions, which are addressed in the following sections.

Section 4 proposes that there are evolutionary reasons to expect an analogue model of space in the brain. Even without consciousness, there are major cognitive and fitness benefits to having an analogue model of local space.

Section 5 of the paper proposes that an analogue model of local space can be stored (as a Fourier transform of physical space) in a wave excitation in the brain; and that the wave is in the mammalian thalamus (Worden, 2020a). It discusses possible reasons why such a wave has not yet been detected.

Section 6 states the projective wave theory of consciousness. The theory proposes that a wave excitation in the thalamus is the sole source of phenomenal consciousness. This would imply that all consciousness is spatial consciousness. The theory says that an analogue model of local space is a necessary condition for consciousness, but not a sufficient one. The analogue model may need to reside in a particular physical substrate, to be sufficient for consciousness.

Section 7 introduces the idea of a ‘Bayesian balance’ to test any theory of consciousness against data about the properties of consciousness. In the Bayesian Philosophy of Science (Sprenger and Hartmann, 2019) any theory can be evaluated by a Bayesian balance between the complexity of its hypothesis, and the data it accounts for.

Section 8 compares the Projective Wave theory with empirical evidence about consciousness. It has a good Bayesian balance—mainly because it accounts well for the geometry of spatial consciousness, which few other theories do. The theory is also compared with six other facets of phenomenal consciousness.

Section 9 compares the projective wave theory with other considerations. The theory gives an account of why consciousness evolved, gives a solution to the decoding problem, and is falsifiable, in that if no wave is ever found in the brain, the theory is incorrect.

Section 10 concludes the paper, discussing the implications of the theory for cognitive science. If this theory is correct, it will replace our current view of the brain (as a complex collection of neurons, and only that) by a nuclear model of the brain, in which all neural computation is centred around a ‘nuclear’ analogue model of local space. This view would be as radical a change as the Rutherford model of the atom, and would open up many new avenues for research.

Theories of consciousness are mainly of two types: theories based on current neuroscience, which assume that consciousness arises from neural firing, and theories which assume some novel mechanism in the brain, such as electromagnetic fields or a quantum mechanical effect. By Occam’s Razor, a novel mechanism can only be justified if it leads to a good fit to the data of consciousness. This paper asserts that a novel mechanism is required, and proposes a mechanism which gives a good fit to the main testable data about consciousness—which is the close geometric match between spatial consciousness and the real world. The good fit to the data is the main justification for the novel hypothesis.

It should be noted that this paper describes an initial conceptual proposal for a theory of consciousness, rather than a fully worked-out theory. There is much detail in the mathematics, and particularly the biophysics of the wave and its coupling to neurons, that remains to be worked out. The concept of consciousness arising from a wave excitation in the thalamus has sufficient potential for agreement with the evidence about spatial consciousness—‘what it is like’—that the theory merits further investigation.

## 2 Computation, brains and consciousness

This section recapitulates the arguments in Worden (2025), which imply that neural computational theories of consciousness cannot be correct.

In any computer, the information which it computes is encoded in a physical form, to enable computation by physical operations. There are many different physical encodings of information, in man-made computers and in brains.

Without decoding the encoded information, you cannot know what the computer is computing about. To decode the information in a computer, you need extra decoding information; that decoding information does not exist inside the computer itself. It exists only in an external representational entity (Horsman and Stepney, 2014).

This implies an ‘Incompleteness Theorem’ for Computers:

The physical events inside a computer do not define what it computes.

This simple statement has profound implications. Its opposite (that physical events in a computer do define a computation) cannot be defended. There are too many clear counter-examples, where the encoding of the information in a computer makes its meaning indeterminate, without external decoding. There is indeterminacy both at the mathematical/syntactic level (what mathematics is being computed?) and at the semantic level (what physical things does the mathematics represent?). Some examples that show this are described in Worden (2025), and a general mathematical analysis is given in Horsman and Stepney (2014).

To give two examples here: A logical AND gate has two input voltages, and one output voltage. If both inputs are 1 volt, the output is 1 volt. If either input is zero volts, the output is zero. So if 1 volt is TRUE, and 0 volts are FALSE, the gate computes a logical AND. However, if you interpret 0 volts as TRUE, 1 volt as FALSE, it is a logical OR gate. The computation it does is not defined by the physical events inside the gate; it depends on how the inputs and outputs are encoded and decoded as Boolean values.

This indeterminacy scales up from a simple logic gate, to any digital computer. If the computer does some arithmetic operation, the arithmetic it does is only defined when you know the number representation—how any number is encoded by a sequence of bits. There is an unlimited number of different number encodings, and we find a few of them in modern computers—binary integers, floating point encodings, and so on. If we interpret some bit string B as meaning a number N, then the computer does an addition. If we interpret the same bit string B as 2**N, then what it does is multiplication. There is an infinite number of ways to read its inputs and outputs. What it computes is not defined inside a digital computer box.

If there is this much indeterminacy even in digital computers—which we have designed and built and understand—there is much more indeterminacy in neuronal computers, where we are only just beginning to understand the encoding used by a few types of neurons—out of many thousands of types of neuron. The mapping from neural spike trains to their meanings is more indeterminate than the mapping from bit strings to their meanings.

The information required to decode (or interpret) the physical events in a computer as a computation resides outside the physical computer, in a so-called representational entity (Stepney and Kendon, 2021). This has profound implications for man-made computers:

1. Inside a computer simulation, what it simulates is not defined. The idea that ‘we could be living in a simulation’ (Bostrom, 2003; Chalmers, 2022) is incorrect.
2. In a running AI application, the physical information is not about any defined thing. All the intelligence lies in human interpretation of the outputs. This undermines the idea of AI ‘superintelligence’ (Bostrom, 2014; Kurzweil, 2024)

The philosophical implications of these results, while challenging, are not the topic of this paper. This paper concerns animal brains and how they are conscious.

If the brain is considered as a neural computer, the physical events in the brain do not define what it is computing about. If we say: ‘the brain is doing computational processes P1, P2, P3, about objects A, B, etc. (in sub-processes P1A, P3B and so on), then that statement is our interpretation of the brain as a computer. Physical events in the brain do not in themselves define computational processes such as P1A. There is no information in the brain to define whether the computational processes are P1A, P3B and so on—or a different set of processes Q5W, Q6Z….

Current theories of consciousness propose that consciousness is caused by physical events in the brain, interpreted as computational processes. I denote these processes by P1, P2…. To take a selection of current theories:

- P1 is ‘representing objects in the visual field with recurrent processes’ (Crick and Koch, 1990; Lamme, 2020)
- P2 is ‘making Bayesian maximum likelihood models of reality’ (e.g. Rudrauf et al., 2017, 2022; Deane, 2021)
- P3 is ‘higher order computation about the brain’s own computations’ (Brown et al., 2019; Lau, 2019, 2022)
- P4 is ‘making information globally available in the brain’ (Baars, 1988, 1997; Dehaene and Changeux, 2011; Dehaene, 2014)
- P5 is ‘linking computational units, with high connectivity’ (Tononi, 2012; Tononi and Koch, 2015; Haun and Tononi, 2019)
- P6 is ‘predictive processing’ (Seth and Hohwy, 2021; Hohwy, 2022)

The difficulty with all these theories is that the computational processes P1. P6 are not a physical property of brains; they are part of our interpretation of brains as computers. That interpretation uses decoding information from outside the brain. We are not entitled to propose, as these theories do, that any P1 or P2 is the cause of consciousness, because in that case, consciousness would depend on events outside the brain—on our external decoding process.

Consciousness contains un-encoded information about objects such as an object A—including information about its spatial boundaries. Any theory which says ‘consciousness of A is caused by process P1A, which is about A’ cannot be correct—because process P1A does not exist inside a computer brain. It exists only in our external decoding of the neural events. Encoded information in a brain cannot give the unencoded information in consciousness.

If the encoding of information in the brain is regarded as encryption, the difficulty is particularly clear. It is obvious that without decryption, encrypted information has no meaning; and that any decryption key resides outside the brain. Therefore in neural computational theories of consciousness, physical events inside the brain do not define the information content of consciousness. Those theories cannot be correct.

To restate this point: even when a computational interpretation is correct, it is not what happens physically in the brain. What happens in the brain is an encrypted message; our interpretation is the decrypted message. The brain itself does not have the decryption key. A computational theory of consciousness cannot decrypt the neural events in the brain, to give clear, unencrypted consciousness.

This result could set off a search for loopholes, to try to refute the result and rescue neural theories of consciousness. There is a simpler alternative—to seek a theory of consciousness which works if the result is correct. If the result is correct, it implies that to account for consciousness, something else is required in the brain, besides neurons firing. This paper proposes a novel mechanism in the brain, that fits the data of consciousness well enough to justify the hypothesis.

## 3 An analogue model of local space

The difficulty for computational theories of consciousness is that a computer contains only encoded information, whereas consciousness contains unencoded information about space—such as the boundaries between spatial regions. In physical space, the edge of a leaf is a boundary between leaf-stuff and air-stuff; in conscious awareness, the same boundary exists, in an un-encoded form, as a boundary between qualia (leaf qualia and empty qualia) in an experienced space. We consciously experience the boundary—not an encoded form of it, such as a symbol. We experience the geometric structure of a boundary, with no encoding.

To account for this conscious experience, we need to find a physical structure in the brain which holds some of the same information as real 3-D space, in an unencoded form. Neurons do not do this, because neural information is highly encoded as firing rates of spike trains (or in other ways) in a topological network of neurons, axons and dendrites. Firing times and network topology can only be an encoded version of space.

In Worden (2025), I suggested that an analogue model of 3 dimensional space—such as the image formed by a lens—holds spatial information which is not encoded. An image of some region formed by a lens has spatial boundaries between sub-regions, corresponding directly to the boundaries in the real region of space. This is unencoded meaning.

This paper proposes that the mammalian brain holds an analogue model of the 3-D space around the animal; and that the neural computer brain exchanges information with the model. This proposal raises questions:

1. Is it physically possible to hold an analogue model of space in the brain?
2. Is it physically possible for neurons in the brain to exchange information with the analogue model?
3. Would such an arrangement contribute to fitness? Is there any reason for it to have evolved?
4. Is nature capable of evolving this mechanism?
5. If such a mechanism has evolved, can it account for what we know about consciousness?

This paper gives answers those questions, answering ‘yes’ to all of them. Much can be said about each question (e.g. Worden, 2020a, 2024b); what follows is a summary.

Addressing question (1): no brain can hold inside it a miniature replica of local 3-D space. That would need to be a dynamic replica model, built and re-built several times per second, as things move in the space around the animal. No such region has ever been detected in a brain; and usually, macroscopic biological matter does not build and rebuild itself several times per second; it would be metabolically very expensive to do so. We require an analogue model of space which is not a direct miniature replica of reality.

An analogue model can be held in a mathematical transform of local space, in a wave excitation. Specifically, the transform could be a Fourier transform of space. In this model, the wave excitation is a superposition of many waves with different three-dimensional wave vectors^1^ **k**—such that the component with a wave vector **k** represents some object at a three-dimensional position **r** = c**k**, where c is a constant.

This is biologically possible, because animal bodies can hold wave excitations inside them—such as sound waves in the vocal tract. A sound wave can be rapidly created and recreated many times per second, as it is made of air (not biological matter); and it can hold large amounts of information, in different wave vectors (we do not expect to find sound waves in the brain; they are used here merely as an illustration).

One might object that a mathematical transform of space is an encoding of space—so the problem of encoding and decoding has not been removed. However, the Fourier transform has a special role in science and physics (for instance, in optics and in quantum mechanics; Feynman et al., 1965). To invert a Fourier transform (to recover a spatial distribution from a wave, as for instance in a hologram), does not require large amounts of decoding information; it is a simple mathematical operation, defined by one numerical scale factor. The Fourier transform is a fundamental part of how we describe nature, in quantum mechanics. The decoding objection does not apply, because almost no decoding information is required (just one scale factor). A Fourier transform is not an encryption.

Just as a wave excitation can be rapidly built and rebuilt in the vocal tract, so it may be possible to rapidly build a wave model of local 3-D space in the brain—providing a possible answer the question (1).

This entails a possible answer to question (2). If the wave excitation occupies some approximately spherical region in the brain, such as the thalamus (Worden, 2020a; Worden et al., 2021), then a single neuron can have many synapses spread throughout the region. The synapses might act as transmitters and receivers for the wave—so each neuron permeating the region can couple selectively to certain wave vectors, representing objects at certain spatial positions. Each neuron can act like a steerable antenna immersed in the wave, selectively exchanging information with the wave. By this mechanism, the wave can act as an efficient switch between large numbers of neurons—delivering high dynamic switching connectivity.

## 4 The primacy of spatial cognition

We next address question (3): if there is an analogue model of space in the brain, coupled to neurons, can it be of any use? Could it contribute to fitness? Is there any selection pressure for a wave to evolve?

A primary requirement for any animal brain is to know where food is, so as not to starve. Here, the important word is ‘where’—the animal needs to know precisely where food is relative to itself, to reach and get it. Spatial cognition is the core of most animal cognition—a vital tool for survival at all moments of the day.

This suggests that if animal brains held a persistent representation of local 3-D space, maintained by integrating sense data of all modalities, that model could have major cognitive benefits (Worden et al., 2021; Worden, 2020b):

- It could act as the single repository for a Bayesian maximum likelihood model (Knill and Pouget, 2004; Worden, 2024c) of the locations of all things in local space (including the animal’s own body, and other nearby objects), got by integrating sense data of all modalities, such as vision and touch.
- Information from this resource could be used to classify local things into the types of thing requiring actions. Things could be classified based on their inferred full 3-D shape, which is a more invariant and reliable classifier than, for instance, a 2-D visual projection of a shape.
- The repository could act as a short-term memory for information derived from sense data, allowing that information to be reused for different purposes, over the time intervals over which it can be expected to be reliable.
- As a hub for all spatial information being processed and used in N different neural regions, the repository could integrate that information using of the order of N neural connections to those regions, rather than requiring N(N-1) neural connections as would be needed for direct peer-to-peer connection between the regions, and allowing efficient parallel iterative integration of sense data (Worden, 2020b).
- As the repository of all information about what is where in space, it can hold the master answer to the vital question ‘where is food?’, allowing the animal to simulate possible motions to get food (and for all other purposes) to anticipate their consequences.

To serve these multiple purposes the repository should store spatial information in a neutral representation, easily encoded and decoded to specialised neural codes used by the different neural regions it connects to. An analogue representation is the most neutral possible representation, minimising the problem of encoding and decoding.

Thus a central analogue model of local space could have considerable cognitive benefits, increasing the efficiency and decreasing the cost of computations vital to the animal’s survival. Understanding what is where in local space is the most important task of an animal’s brain, and an analogue model of space (if it has evolved) is a major enabler of this central function.

In recent years animal brains have been shown to use Bayesian cognition in many domains (Rao et al., 2002; Knill and Pouget, 2004; Friston et al., 2006; Friston, 2010; Friston et al., 2017). The central problem of Bayesian cognition is to build and maintain a Bayesian maximum likelihood model of the positions and nature of all local objects, integrating sense data of all modalities—to choose any possible action using the very best bet about what things are where. An analogue model of local space can be the hub for this most vital of all Bayesian inference tasks.

A wave representation of local space has a further great benefit over any purely neural representation: combining high precision with high speed (Worden, 2020a). If locations in space are represented by neural firing rates, in a period T which is sufficient for S neural spikes, because of statistical noise the precision achievable in any spatial coordinate is approximately one part in √S. The animal’s model of nearby space needs to be updated over very short timescales, of the order T = 1/5 s. In this timescale, the maximum number of neural spikes S is typically of the order 100. More typical firing rates give S = 10, giving a precision of only one part in 3. This precision is much less than the spatial precision of sense data such as vision or touch; it seems unlikely that animals would have evolved precise sense data, if that precision could not all be used in the internal representation of space.

A wave representation of space can be updated within a small fraction of a second, yet it can have high spatial precision, of the order of one part in W, where W is the number of wavelengths of the wave excitation which fit into the region of the wave. If the wave is in the thalamus, its minimum wavelength can be very small, making W as large as 50 or more, even for small mammals. This combination of speed and precision is much better than any purely neural storage can give. The importance of this practical engineering benefit of a wave can hardly be over-estimated.

There is a further feature required of the wave analogue model of space. In such a wave excitation, there is some minimum possible wavelength—equivalently, a largest possible wave vector **k**. In a direct Fourier transform of space, a wave with wave vector **k** represents an object at position **r** = c**k**, where c is a constant. Animals need a model of space which can represent objects at any distance—effectively, up to infinite distances. How can a wave with only limited wave vectors represent very large distances?

One solution is for the wave not to represent 3-D space directly, but to represent some mathematical transform of space, which brings infinite distances down to finite distances. Ideally the transform would introduce as little geometric distortion as possible—because any spatial distortion makes computation more complex. The best choice may be a projective transform, as in Projective Geometry, which brings objects at infinite distance down to a finite distance (which the wave can represent) but which introduces very little distortion (Rudrauf et al., 2017). For instance, straight lines in real space are still straight lines in projected space. I therefore assume that the wave in the brain holds a projective transform of physical space. The wave is a Fourier transform of a projective transform.

In summary, an analogue model of space, held in the brain in a wave excitation, can have major evolutionary and engineering benefits, efficiently supporting the core function of Bayesian spatial cognition. If it is physically possible to do this, there are good reasons for it to have evolved.

## 5 Is there a wave in the thalamus?

I next address question (4)—can wave in the brain have evolved? The leading objection to the idea of a wave excitation holding spatial information in the brain is that no such wave has yet been detected. I suggest that this is not a strong objection.

If there is an analogue model of space in the brain, it is likely to be old in evolutionary terms (dating back as far as the Cambrian era, when animals first had complex sense data), in one of the older regions of the brain (Merker, 2007). It is also expected to be well connected to diverse sensory and motor regions of the brain, to integrate spatial information from all of them. A leading candidate region to hold the wave, in mammals and other vertebrates, is the thalamus (Worden, 2020a). The thalamus has strong reciprocal connections to many brain regions, as is required to exchange spatial information with them.

Although a wave has not been detected in the thalamus, there is indirect evidence for a wave. In a purely neural synaptic model of the thalamus (the alternative to a wave), the anatomy of the thalamus does not make sense. The mammalian thalamus has many nuclei, with weak or non-existent connections between them (Sherman and Guillery, 2006). Why then are the nuclei clustered together in a central location in the brain? The same neural connections could be made using less brain energy (with shorter axons) if the thalamic nuclei were to separate and migrate outwards towards the cortex (Worden, 2010, 2014). The anatomy of the thalamus only makes sense if there is something else going on, as well as neural synaptic connections. If there is a wave, all the thalamic nuclei need to be clustered together, to be immersed in the same wave. A wave makes sense of the anatomy of the thalamus.

Another significant feature is the highly preserved near-round shape of the thalamus, in all mammalian species (Sherman and Guillery, 2006; Jones, 2007; Hailey and Krubitzer, 2019)—a big contrast to the variable and contorted shapes of other brain regions, such as the cortex or the hippocampus. Why is the thalamus always nearly round? A near-spherical shape is well-suited for holding a wave excitation—with similar ranges of wave vectors available in all three spatial dimensions, to represent objects at any location around the animal.

Energy consumption is a possible reason why no wave has yet been detected in the thalamus. If 3-D space is represented by a wave, the wave needs to be active at every moment of the day. To reduce its energy consumption, the wave needs to have as low intensity as possible, while still being able to couple to neurons. Neurons can couple to wave excitations of very low intensity (for instance, they can couple to light, down to the one-photon level), so we would expect the wave in the thalamus to have extremely low intensity, and therefore be hard to detect. We have not yet detected a wave in the thalamus, simply because we have not yet looked for it with the right detectors.

There are electromagnetic fields in the brain, possibly with a wave-like spatial dependence (Pockett, 2000; McFadden, 2002). However, these fields are a passive trace of neuronal electrical activity. Hence they do not store information for any appreciable time, and cannot act as a persistent store of spatial information—as a short-term memory. This implies that the wave in the thalamus is probably not an electromagnetic wave. That is another reason why it has not yet been detected, because most detectors of brain activity are electric or magnetic detectors.

There is no shortage of other candidates for a thalamic wave, similar to the many quantised excitations which exist in solids. Finding a possible substrate for the wave in the brain is a very challenging problem in biophysics, which needs to be addressed before any direct attempts to detect the wave.

There may also be a wave in the invertebrate brain, with a similar spatial role to the wave in the thalamus. The central body of the insect brain, like the mammalian thalamus, shows strong indirect evidence of being the site of a wave excitation, storing spatial information (Worden, 2024a). The insect central body has neural connections to a wide range of sensory and motor areas, and is linked with spatial cognition (Strausfeld, 2011; Chittka, 2022). The shape of the central body (the fan-shaped body and the elliptical body) is remarkably conserved across all insect species, and is an approximately round shape (Heinze et al., 2021), which is well suited to hold a wave in three dimensions. Invariant across all insect species, this is a highly significant clue that something besides neural synaptic connection is happening in the central body; and that the something could be a wave. Flying insects need sophisticated spatial cognition—and insect brains, being simpler than mammalian brains, may be a suitable place to explore the wave hypothesis.

In summary, there are possible physical mechanisms and anatomical locations for a wave excitation in the vertebrate brain, and in the insect brain. There is some evidence, albeit indirect, that both the thalamus and the insect central body are the sites of a wave excitation. The physical substrate of the wave may be some exotic state that has not yet been studied; but we should not underestimate the ability of evolution to discover and exploit such phenomena.

## 6 The projective wave theory of consciousness

The projective wave theory of consciousness proposes that there is a wave in the mammalian thalamus, which serves as a short-term memory for 3-D spatial information. The wave is a Fourier transform analogue model of projectively transformed 3-D space. The theory proposes that the wave is the sole source of phenomenal spatial consciousness. Neural activity in the brain is not linked directly to consciousness; it is only linked indirectly, through the wave.

A three-dimensional Fourier transform F converts some function f(**x**) of a three-dimensional vector **x** to a function g(**k**) of a three-dimensional wave vector **k**:


$$g(k)=F(f(x))=\int d^{3}x\;\exp.(2\pi i\;k.x).$$ (1)

The transform can be inverted by using the same integral, with (i) replaced by (−i) and the integral d^3^**x** replaced by d^3^**k**. The Fourier transform is the mathematical basis of holograms.

A projective transform P of a three-vector **x** defines another three-vector:


$$x^{’}=P(x).$$ (2)

P is defined by embedding **x** in a four-dimensional projective space, applying a linear matrix transformation in that space, then recovering the three dimensional result vector **x**’ by division, inverting the embedding. Details are given in Hartshorne (2009).

The theory proposes that the wave in the thalamus is made by first applying a projective transform to locations of objects in real external space, then applying a Fourier transform. The projective transform brings in all objects at large distances to within some limited distance, which transform to finite wave vectors **k** (i.e., to non-zero wavelengths):


$$g(k)=F(P(x))\;\text{with}\;\mathrm{all}|k|<k_{\max}.$$ (3)

Here, F and P are as defined in Equations 1, 2. Equation (3) states in mathematical terms what is claimed by the theory, and how that claim relates to the Fourier transform F and the projective transform P.

Physically, this is done by neurons having wave transducers distributed across thalamic nuclei. The transducers of any one neuron are distributed across its axons and dendrites in a sinusoidal, wave-like manner, with a Gaussian cutoff at high spatial frequencies, so that the neuron couples selectively to a narrow range of wave vectors. The transducers (whose nature is currently not known, but which could be in compound synapses) couple to the wave both as transmitters and receivers. Neurons use the wave as a short-term memory for the spatial locations of objects, inferred from sense data.

In using a Fourier transform of 3-D space, the mathematical model of how neurons couple to the wave is similar to the theory of Segman (2020, 2024). It appears not to be identical, and further investigation will be required.

This paper is an initial conceptual proposal for a theory of consciousness, rather than a fully worked out theory. Much detail of the mathematics and biophysics of the wave remains to be worked out; so a detailed comparison withn Segman’s theory is not yet possible.

In this theory, a wave excitation holding an analogue model of local 3-D space is a necessary condition for phenomenal consciousness; but it is not a sufficient condition (if any kind of wave were sufficient, we could already create consciousness in the lab, using existing technology).

I suggest that to give a sufficient condition for consciousness, the wave is required to have some specific physical nature and substrate. Any old wave, such as a sound wave, would not be conscious. Because the theory does not propose a sufficient condition for consciousness, it does not claim to solve the Hard Problem of Consciousness (Chalmers, 1995, 1996). It may be some time before we know enough biophysics of the brain to confidently propose a physical substrate for the wave. Such a proposal would be a step towards solving the hard problem—leading possibly to a different hard problem (just as in physics, there is a regress of problems; each new ‘fundamental’ constituent of matter—atom, proton, quark,…—leads to a further problem: what are its constituents? The infinite regress of problems in physics is a hard problem, never to be solved).

While this theory says little about the existence of consciousness, it says a lot about the properties of consciousness; so it is a testable theory. Mainly, it makes predictions about the geometric properties of spatial consciousness. It says that spatial consciousness of things around us in the present moment arises from the wave, through a Fourier transform (the wave is like a holographic model of reality). So the theory makes clear predictions for the geometric properties of spatial consciousness—predicting that geometrically, consciously perceived space is very similar to real physical space.

The theory makes another prediction, which may at first seem disturbing. It predicts that spatial consciousness is the only consciousness there is. There is no other consciousness. You may then ask: what about thoughts, emotions, sounds, and feelings? The theory says that these are qualia, and that all qualia are located in one consciously experienced space. I suggest that this is actually consistent with our conscious experience, as discussed in section 8.

The predictions of the theory are compared with evidence about consciousness in the next three sections.

## 7 Testing a theory of consciousness: the Bayesian balance

How can we assess the projective wave theory, and compare it to other theories of consciousness?

An accepted process to evaluate a scientific hypothesis is to calculate the probability that it is correct within its domain of applicability, as a Bayesian posterior probability (Sprenger and Hartmann, 2019). Any hypothesis has only a small initial (prior) probability of being correct, and that prior probability is modified by Bayes’ theorem in the light of its fit to experimental data, to give a posterior probability. Only if the posterior probability is near 1.0 can we believe the hypothesis, and start to call it a theory.

This analysis is how all theories are evaluated—by their fit to experimental data. Some established theories of physics and chemistry agree well with an enormous amount of data, using only simple hypotheses; so they have a massively positive Bayesian balance. Their Bayesian posterior probability is very near 1.0, and they are taken to be correct within their domains of application.

All theories and data are approximate. The truth of a theory is not affected by data from outside the domain where its approximations apply. So, for instance, Newtonian dynamics has not been rendered untrue by special relativity or quantum mechanics; they have different approximations, applicable in different domains.

From this viewpoint, where do theories of consciousness stand? They are hypotheses rather than theories. They can be assessed by a Bayesian Balance between two terms:

- Hypothesis complexity: How much information (e.g., in bits) is needed to state the hypothesis?
- Agreement with data: how significant is the agreement between the hypothesis and empirical data (agreement can also be measured in bits)?

Bayes’ theorem can be used to calculate a balance between these, where each side is approximately the logarithm of a probability, measured in bits. Hypothesis complexity is the debit side, and agreement with the data is the credit side. Only when a hypothesis has a positive balance of credit over debit can our level of belief in it come close to 1.0.

**Figure 1:**
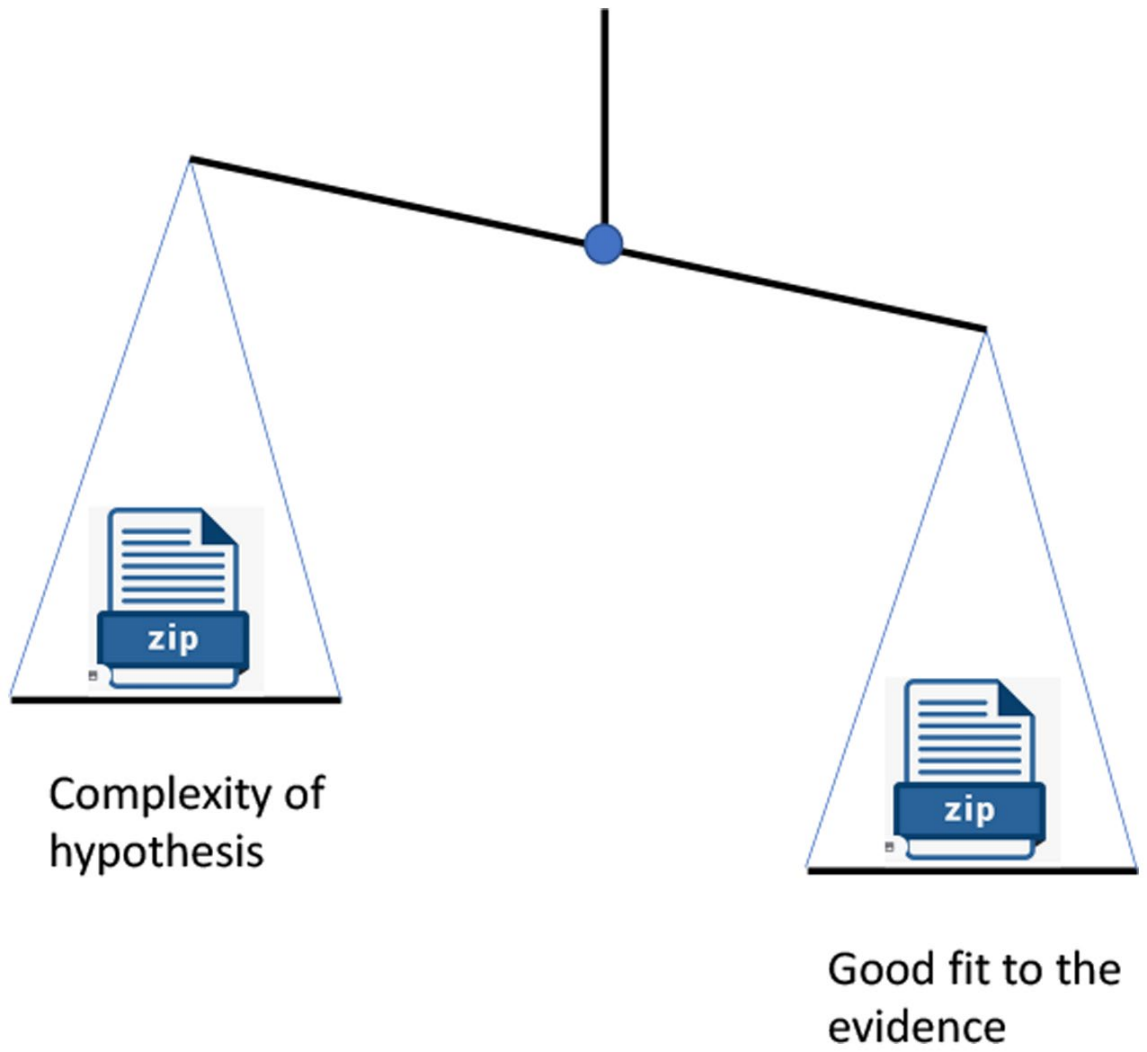
The Bayesian balance for a successful theory. The information content of the evidence fitted by a theory, as measured by the zip file test, must be greater than the information content of the hypothesis.

The Bayesian Balance of theories is illustrated in Figure 1.

The two sides of the Bayesian balance can be informally measured by a ‘Zip file test’. This test is to collect a statement of the hypothesis, and a statement of the evidence it agrees with, in separate text files, and compress both files using a zip compression tool. The sizes of the resulting zip files (measured in bits) are an approximate measure of the information content of each side—the sides of the Bayesian balance.

There is not space here for a detailed assessment of the ‘complexity of hypothesis’ side of the balance for this theory of consciousness, or for others. In any case, that is the less important of the two sides of the balance; whatever the complexity of its hypothesis, any theory needs a good fit to the evidence before it can be believed. I only note that qualitatively, by making consciousness depend only on one wave excitation (rather than on complex properties of large neural circuits) the projective wave theory has a virtue of simplicity; a small debit side to its balance. However, much detail of the maths and biophysics of the theory remains to be worked out.

The Bayesian balance is the quantitative statement of Occam’s Razor. Any new hypothesis adds to the debit side of the Bayesian balance. It is only justified if it improves the fit to the data—adding enough to the credit side, to offset what it has added to the debit side.

I next turn to the ‘fit to the evidence’ credit side of the balance.

Chalmers (1996) has discussed the kinds of evidence available to test a theory of consciousness. We need to discuss phenomenal consciousness (the ‘what it is like’ to be alive; Nagel, 1974; which Chalmers calls conscious experience of something) rather than access consciousness (Block, 1978; which Chalmers calls awareness—the ability to act on some piece of information, or to report it verbally). There is a strong correspondence between access consciousness and phenomenal consciousness, and we use access consciousness as evidence about phenomenal consciousness. There are philosophical concerns about these issues, going as far as solipsism; but if you consistently applied a skeptical philosophical strategy to deny the validity of data, then no science would be possible. Without some realism, consciousness (or any other natural phenomenon) is not an issue, and there would be no need to make theories of it.

## 8 Comparing the projective wave theory with evidence

I shall discuss several types of evidence about phenomenal consciousness, which can be used to test any theory:

1. Geometric shapes of things in conscious awareness, derived from sense data at the present moment.
2. Consciousness of imagined things and remembered things
3. Consciousness of thoughts, inner monologues and bodily feelings
4. Consciousness of self (both basic and higher-order consciousness of self)
5. Diversity of qualia, and similarity scales of qualia (colours, sounds, smells…)
6. Anomalies and illusions of consciousness
7. Levels of consciousness—how they vary over time, depending on physical conditions

Item (1) may a richer source of testable data than the other categories, in its potential to tip scales of the Bayesian balance. If at any moment we ask: ‘what is my conscious experience now?’, a key part of the answer is ‘I am aware of the things around me’. In particular, I am aware of their geometric dispositions in three dimensions. That conscious geometric information has high information content—possibly as large as thousands of bits of information at any moment.

For instance, you experience the edge of your desk as a straight line, with some high level of precision. Precision is information content; you could detect deviations from a straight line at the level of one part in 50 (2^−6^, or 6 bits) in any of 10 regions along the edge—which makes 60 bits, just from one small aspect of your experience. There are many other aspects of conscious experience in any short time interval, as your attention goes to different things. There is a lot of information about the general disposition of things in space; the geometry of your conscious awareness matches the geometry of real space, at all scales between the finest scale near your centre of view, and the whole of your conscious space. This is a lot of matching.

I suggest that the information content of this data (the integrated geometric match between conscious experience and real physical space) is of the order of 1,000 bits in any second—in any case, not as small as 100 bits, and not as large as 10,000 bits. In terms of the Bayesian balance, point (1) of the list above has much greater potential than all other items in the list.

The testable fact is that the geometry of your conscious experience matches the geometry of real space around you, in some respects with high precision. This is testable. You can take a ruler, a taut string, or a set square, and verify the geometric match. For instance, you can test that what you experience as a straight line really is a straight line, by its mathematical definition—the shortest distance between points, as tested by a taut thread. This testable geometric match is something that a theory of consciousness can predict.

The projective wave theory predicts in a natural and parameter-free way that the geometric properties of spatial experience closely match the geometric properties of real physical space. This agreement contributes something of the order of 1,000 bits to its positive Bayesian balance. The same cannot be said for many other theories of consciousness—many of which are silent about the spatial geometric nature of consciousness. For instance, if consciousness of space arises from a highly distorted visual cortex, why is consciousness spatially undistorted? Many neural theories of consciousness do not address this question, although some do (e.g. Haun and Tononi, 2019).

So the projective wave theory does well on item (1) above—which is by far the largest term in the Bayesian balance.

To compare the projective wave theory with other data about consciousness in items (2)–(7), we need to consider the role which the wave plays in spatial cognition, as discussed in section 4. The wave-based analogue model of space is a hub for the integration of multi-modal sense data in a single maximum likelihood Bayesian model of local space. The wave integrates sense data of all modalities—including vision, sound, touch and other bodily feelings—in a single model of space. This predicts that consciousness, derived from the model, includes consciousness of sounds, touch, and other bodily feelings. All these are experienced as qualia located in an experienced space.

This prediction of the theory agrees well with our conscious experience. For instance, we experience located sounds and located bodily feelings. The distinctive prediction of the theory—that all consciousness is spatial consciousness—is not a difficulty, but agrees with the evidence.

Section 4 described two roles in spatial cognition for the analogue model of space:

- To compare the spatial configuration of the present moment, with the spatial configuration of some previous similar moment (such as a memory of what happened in the past at the same place) to anticipate what may happen next.
- To simulate some possible physical action, to anticipate its consequences (such as ‘can I jump over that rock?’)

These both require a capacity to drive the analogue model not only from current sense data—to experience reality—but also to drive it from alternative versions of reality—to experience memories or imagination.

Since there is only one analogue model of space, both reality and imagination need to be experienced in the same conscious space. There is a biological need not to confuse real things with imagined things (not to hallucinate) so imagination is experienced as a kind of pale, transparent ‘ghost’ superimposed on current reality. Imagination is less vivid than experience of reality, for two good reasons—first, because fewer neural resources are devoted to creating it in the analogue model; and second, so it does not interfere with the more essential task of modelling of reality (i.e., so as not to hallucinate).

In these respects, the projective wave model agrees with our conscious experience. We experience one space containing both current reality and imagination (this is the unity of consciousness, arising from a single wave excitation); and we experience imagined things, weakly superimposed on reality.

What about item (3), consciousness of thought? We experience conscious verbal thought as the sound of imagined words, located somewhere inside our heads. This is what the projective wave model predicts—thoughts are imagined sounds located inside the head, which are used to plan future speech and actions. The experienced location of thoughts is an empirical fact about consciousness.

Emotions are experienced as feelings located in the body, together with the sounds of some inner verbal commentary on events. The projective wave model accommodates the conscious experience of emotions.

For consciousness of self, item (4), there is some difficulty in pinning down just what is the conscious experience of self. If we cannot pin down the evidence, it is hard to know what contribution it makes to the Bayesian balance of a theory. One hypothesis is that the full sense of self is a peculiarly human attribute, not shared by other species, and that it derives largely from language. In order to converse with others, we need to infer what they are thinking about us; so the human sense of self (and our self-esteem, which drives many emotional feelings) evolved recently to support language. In this view, consciousness of self is nothing more than consciousness of the verbal thoughts and bodily feelings arising from our language-based sense of ourselves; and it raises no new issues for the projective wave theory. This analysis applies both to basic consciousness of self (however that is defined), and to higher-order consciousness of self (which could be a language-derived phenomenon).

Some people may be uncomfortable with this analysis; a question for them is: what actual data makes them uncomfortable? Or is it merely an intuition which feels to be violated?

Item (5) is the diversity of qualia—that we experience many different qualia for sounds, sights, and so on. All qualia are experienced in some region in our consciously experienced space. In terms of the projective wave theory, a spatial location is a wave vector. So for any single wave vector, the wave excitation must have many degrees of freedom, to support our experience of diverse qualia at any single location.

Continuously varying qualia, with similarity scales between them, arise in a straightforward way from mixing of a few basic qualia—just as all colours can be made by mixing a few primary colours. In the projective wave model, the wave at any location (at any wave vector) can be an arbitrary mixture of certain basic components, which are the degrees of freedom of the wave, just as a mixture of primary colours can make any colour.

Another empirical fact to be accounted for is that while different people’s qualia may differ (as qualia are not in the common ground; Tomasello, 2014; between people, which underpins language, so there are things that cannot be said about them). We can discuss similarity scales of qualia, which are in the common ground, and find agreement. This fact does not pose problems for the projective wave theory; it is more a fact about how language works.

The requirement for diverse qualia—for many degrees of freedom in the wave—is a demanding requirement on the biophysics of the wave. It implies, for instance, that the wave cannot be an electromagnetic wave, because an electromagnetic wave has only two degrees of freedom, and we experience more than two dimensions of qualia. Electromagnetic waves have two degrees of freedom because photons (the quanta of the wave) have spin 1. This implies that the basic quanta of the wave in the brain must have higher spin than 1—more than 2 degrees of freedom. That is not an impossible requirement, but it requires some ingenious design by evolution—possibly starting from a low-spin wave, and evolving incrementally to higher spins, to make the spatial model more discriminating, with more degrees of freedom, and so more independent qualia. Having more qualia gives fitness benefits, as the wave model of space has greater capacity.

I shall say little here about item (6)—anomalies or illusions of consciousness, such as out-of-body experiences, or the moon illusion. While they are interesting, I do not think they make a large contribution to the Bayesian balance of a theory, compared to everyday spatial consciousness, item (1). I know of no reasons why the projective wave theory cannot account for anomalies of consciousness.

Amongst the most interesting ‘anomalous’ (meaning, infrequent) states of consciousness are the many reports of mindful states of consciousness, notably in Metzinger (2021). I do not think those reports constitute evidence against this theory; they are a fascinating input to the theory, and it is not possible to do justice to them in this paper. Analysing those states of consciousness in this theory is a whole other research project, and possibly one of great importance.

Under item (7), there is some available information about levels of consciousness in states such as wakefulness, sleep, or coma. These data are slowly varying in time, and have small information content—so in the Bayesian balance of a theory, they do not contribute much. They arguably depend on a secondary question—what are the consequences of a theory of consciousness in abnormal conditions—which may be addressed after the theory has been compared with the primary data of consciousness in normal conditions.

That completes the discussion of the seven empirical properties of conscious experience, listed at the start of this section. In summary, the projective wave theory has a good Bayesian balance, largely because of its good agreement with the first item (the spatial form of consciousness), which gives by far the largest contribution to the Bayesian balance; and I know of no major difficulties in addressing the other items.

## 9 Other criteria for a theory

As well as these comparisons with empirical data, there are other questions which enter into our assessment of a theory of consciousness. These questions include:

1. Why consciousness evolved, and why it has the properties it does.
2. The problem that neural information in the brain is highly encoded—unlike spatial consciousness, which is an unencoded model of 3-D space.
3. The existence of consciousness (the hard problem)
4. A bio-physical mechanism of consciousness, possibly linked to the avoidance of pan-psychism
5. The unity of consciousness
6. The time delay between physical events and our conscious experience of them
7. The timescales over which conscious experience changes
8. The ability to focus selective attention on aspects or regions of consciousness

I discuss these in turn.

Under item (1), evolution can pose a problem for neural theories of consciousness. In many theories, consciousness is an epiphenomenon of neural activity; although neurons cause consciousness, consciousness has no causal effect on neurons. If consciousness does not alter cognition, behaviour, or fitness, there is no selection pressure to have made consciousness evolve to its present form. This would mean that the remarkable realistic form of consciousness is just a huge coincidence, not shaped by evolution. Requiring such a large coincidence adds a large amount to the debit side of the Bayesian balance of a theory, making the theory much less credible. It goes against a dictum which holds for all the rest of biology: that nothing in biology makes sense, except in the light of evolution—that all design in biology is the result of evolution. We would prefer consciousness to be a result of evolution.

The alternative for neural theories of consciousness is equally unattractive. If consciousness has some causal effect on neural firing (so consciousness can have evolved under selection pressures), its causal effect has to be non-local—in that through the intermediary of consciousness, any neuron in the brain can have non-local causal effects on any other neuron. Such a non-local effect would take us outside the domain of classical dynamics. Without classical locality, hypotheses are generally highly unconstrained, and so are untestable.^2^ There is currently no suggestion for how a non-local theory of consciousness could be made constrained and testable.

In the projective wave theory, consciousness emerges from a wave in the brain, and there are sound evolutionary reasons for the wave to have evolved to its present form. The wave serves a vital function in supporting spatial cognition, and there has been strong and sustained selection pressure to make the wave take the form it has—a faithful geometric model of reality. Through these selection pressures, spatial consciousness has evolved to its present form; no coincidence is required.

For item (2): as described in section 2 of this paper, a major difficulty for all neural theories of consciousness is that in neural computational models of the brain, spatial information is heavily encoded as neural firing rates, neural synchrony, and so on—whereas conscious experience contains information about space which is not encoded; in neural theories, consciousness requires decoding. The information required to decode neural information does not reside inside the brain; so in those theories, there is no way for physical events inside the brain to cause spatial consciousness, as we experience it. I suggest that this is a very serious difficulty for those theories (Worden, 2025). The projective wave theory avoids this difficulty, by proposing an analogue model of local space in the brain. The analogue model, which needs no decoding, is the sole source of consciousness. This avoids the decoding problem.

For item (3), the Projective Wave theory does not solve the hard problem of why there is any consciousness at all (Chalmers, 1995, 1996). In this theory, a wave in the brain is seen as a necessary condition for consciousness to exist, but not a sufficient condition. I do not think this is a big drawback of the theory. There are other hard problems in science (such as: why does the universe exist? Why do quantum states exist? What is the most fundamental particle?). These problems have not hindered progress in physics. Physics has looked for testable theories of the properties of things, rather than the existence of things. Any testable theory must ultimately rest on some deeper theory, in a way that threatens an infinite regress. But science has to stop somewhere. In practice it stops when we cannot get any more data to test deeper theories. Science is a journey; it is a matter of making progress and accounting for the experiments we can do; not of finding some ultimate reality.

For item (4), we can ask: if a wave in the brain is only a necessary condition for consciousness, what might be a sufficient condition? If we allowed any old wave in the brain to be the seat of consciousness, this would lead to an unattractive form of panpsychism. It would make any wave excitation (such as wave on the ocean) a possible source of consciousness.

A sufficient condition for consciousness might be that a wave must have some specific bio-physical basis, for it to be conscious. Cashing out this condition requires advances in the biophysics of the brain, which might not be made soon. The biophysics of the wave, and how it might couple to neurons, is the main area where the details of the theory remain to be worked out, and it is a very challenging research topic. Meanwhile, here is a tentative proposal:

To minimise the metabolic energy costs of the wave, it will have evolved to as low an intensity as possible, while still coupling to neurons, and not being overwhelmed by thermal noise in the brain. Coupling to neurons may not be the limiting factor; what about thermal noise?

There is one rare state of matter, which can hold macroscopic amounts of information, in a way that is immune to thermal noise—as quantum excitations, which have an unlimited lifetime. This state is a Bose-Einstein Condensate (BEC; Bose, 1924; Einstein, 1925; Pitaevskii and Stringari, 2003), such as a superconductor or superfluid. A high -temperature BEC in the brain (Marshall, 1989; Worden, 1999) may seem like a tall order for evolution, but we should not under-estimate the ingenuity of evolution. We might propose—as an adjunct to the projective wave theory—that the wave excitation is an excitation of a BEC in the brain. The BEC would be a sufficient condition for consciousness.

Note that there are objections to the idea of an analogue representation in classical dynamics, as any classical system can be indefinitely sub-divided into smaller parts, which, it could be argued, are encoded. These objections do not apply to a quantum state such as a BEC, which is indivisible.

This would predict that consciousness only occurs in BECs—a very rare state of matter, which avoids a risk of rampant panpsychism. It aligns well with BECs’ low energy consumption, and their ability to hold information for long periods. There have been arguments against coherent quantum effects in the brain, based on fast quantum decoherence (Schlosshauer, 2010; Tegmark, 1999; Koch and Hepp, 2006). However, information in a BEC is immune to quantum decoherence; BECs behave like long-lived quantum states (Feynman et al., 1965). BEC-like states in biological matter have been investigated by (Frohlich, 1968). There is also emerging evidence for quantum effects in the brain (Li et al., 2018; Kerskens and Perez, 2022).

While it is known that neurons can couple to some waves (such as light) at very low intensity, it is not known how they might couple to waves in a BEC. If the idea of a BEC wave gains support, this would be a topic for future research.

For item (5), we experience a unity of consciousness, which is hard to define in words, but is an internal reality of the mind. This may be a difficulty for neural theories. Neurons in brains can do many things in parallel; why not parallel consciousness? The unity of consciousness poses no difficulty for the projective wave theory. In this theory, there is only one wave in the brain, located in the thalamus. As the wave is the source of consciousness, there can be only one consciousness, in one experienced space—containing all types of qualia, and containing both reality and imagination (with all qualia forced to play on the same stage). The unity of consciousness is a simple consequence of the projective wave theory.

On item (6), it is known that conscious reality of the present moment (including deliberate actions) follows shortly after the physical events that it describes—by an interval of the order of 1/3 s (Libet et al., 1983). This is as expected in the projective wave theory. The role of the wave is to hold a Bayesian maximum likelihood model of local 3-D space in the present moment—or as close to the present moment as possible. This model cannot be built instantly from sense data, but requires some iterative optimisation, to find the best fit to all sense data. It is plausible in neural terms that this iteration should take of the order of 1/3 s, allowing for iterative trial and error fitting. Consciousness is a maximum likelihood consistent model of reality, 0.3 s ago—because the brain cannot build the model any faster. Consciousness has a temporal ‘thickness’ of the order of 0.1 s, because sense data arrives in the brain only at finite rates; some time is needed to gather enough information to integrate in a model.

For item (7), our conscious experience appears to change over short timescales, of a fraction of a second; not, for instance, in milliseconds or in minutes. This accords with the claim of the theory, that consciousness arises not from neural processing (which changes very rapidly, over timescales less than 100 milliseconds), but from a spatial memory, which persists over timescales of the order of 1/3 s. The timescale of change of consciousness matches the timescale of change required for a spatial short-term memory.

Items (6) and (7), both concerning sub-second timescales, raise another issue: the speed and spatial precision of consciousness. Within a sub-second timescale, we can consciously identify features with high spatial precision—typically better than one part in 100. If consciousness arose from a neural representation of 3-D space, this would not be possible, because representation of any spatial coordinate by neural firing involves a hard tradeoff between speed and precision. A precision of one part in 100 could not be attained in 1/3 s, because there are not enough neural spikes in that interval; random stochastic spike noise prevents high precision. However, if space is represented by a wave excitation in the thalamus, that combination of speed and precision is achievable.

Finally, on item (8), we can selectively steer the focus of our conscious attention, typically to regions of our consciously experienced space. This property fits well with the role of the analogue model of space. If that model is used to plan and guide physical actions (as it is for most animals), some spatial regions of the model are expected to temporarily have greater importance than other regions—requiring greater neural resources to compute them, to ensure their greater fidelity to reality. These greater neural computational inputs are experienced as a localised increased clarity in the spatial model, which we call attention.

Summarising these eight topics, the projective wave theory has two major benefits over other theories of consciousness, in that (a) it identifies how consciousness evolved, and (b) it avoids the serious decoding problem of neural theories of consciousness. For the remaining six topics, the projective wave theory gives a possible account, and encounters no major obstacles.

## 10 Conclusion and implications for cognitive science

This paper has described in conceptual outline a Projective Wave theory in which phenomenal consciousness arises solely from a wave excitation in the thalamus. The function of the wave is to hold an analogue model of local space—a Bayesian maximum likelihood model, got by best-fit integration of sense data of all modalities. Many details of the theory remain to be worked out.

In sections 8 and 9, I compared the theory with empirical evidence of consciousness, and with other considerations. The theory has a positive Bayesian balance between the complexity of its hypotheses and the evidence it accounts for (particularly the spatial geometric form of consciousness). So it succeeds, by the usual criterion by which the success of a theory is judged. Few other theories of consciousness can claim such a positive Bayesian balance.

Two other merits of the theory are that it gives an account of the evolution of consciousness, and it avoids the serious decoding problem of many neural theories. For these reasons, the theory merits further consideration.

The theory has another major advantage—it is testable and falsifiable. Increasing knowledge of the neurophysiology of the brain, and biophysical models of the wave, will in time lead to stringent tests of the wave hypothesis, both in mammals and (perhaps more easily) in insects. In these more stringent tests, we might find that there is no wave in the brain. In that case, the theory is simply wrong, and we can move on to other theories. That would be progress.

The theory of this paper has some similarities to electromagnetic theories of consciousness, and readers may ask: Could the wave in this theory be an electromagnetic wave? This theory differs from E-M wave theories of consciousness [surveyed by Jones and Hunt (2023)] in several respects:

1. Fit to the data of consciousness: The first task for a theory of consciousness is not to satisfy philosophical preferences, or to propose new mechanisms; it is to fit the data about consciousness. The only rationale for introducing a new mechanism is if it gives a good fit to the data. The most information-rich data about phenomenal consciousness is its spatial geometric form, closely resembling to the world around us. The projective wave theory has a close and natural fit to that data; the wave in the thalamus is biologically required to be a faithful 3-D model of local space, just as spatial consciousness is. E-M theories of consciousness do not give this natural fit to the data of spatial consciousness; there is not a strong reason for E-M waves in the brain to have any particular form. The diverse folding of brain tissue in different species and in different sensory areas suggests that E-M fields in the brain have no particular spatial form or resemblance to external 3-D space. Consequently the hypothesis that an E-M wave is the source of consciousness is not supported by such a good fit to the data of spatial consciousness.
2. The Selection Problem: There are electromagnetic fields in all parts of the brain, such as the cerebellum, which has no known link to consciousness. E-M theories of consciousness have no good criterion to select which E-M fields in the brain are the source of consciousness, and which are not. By contrast, in the Projective Wave Theory there is only one wave excitation in the thalamus, which is assumed to have a special physical substrate (such as a BEC, a very rare state of matter); and that wave is assumed to be the only physical phenomenon related to consciousness. Then there is no selection problem.
3. The Purpose and Evolution of Consciousness: The electromagnetic fields in the brain are largely a side-effect of neural firing, and may not have been designed to serve any central evolutionary purpose. Because of the great speed of light, within the space of a brain E-M fields cannot act directly as a memory for appreciable times—even if they may play some role in memory and attention through neural synchrony (Hunt and Jones, 2023). By contrast, BECs are known experimentally to hold macroscopic amounts of information for macroscopic times; therefore they are a good substrate for a spatial short-term memory, a vital function needed to build Bayesian maximum likelihood spatial models of the local world. In the projective wave theory, there is a strong reason for the source of consciousness to have evolved to this form.
4. Qualia and Shapes in Consciousness: In some E-M theories of consciousness, the form of the E-M field is related to qualia such as smell or colour. In others, the shape of the E-M field is related to geometric shapes in spatial consciousness. But an E-M field cannot represent both qualia and shapes at the same time. If an E-M field represents shapes geometrically (either directly or through a Fourier transform), it cannot represent more than two qualia, because it has only two degrees of freedom (its polarisation). But if the wave is not constrained to be electromagnetic, it can be assumed to have as many degrees of freedom as are required by the space of our known qualia. This assumption could only be justified by a good fit to the data of consciousness, which it has.
5. Panpsychism: Since E-M fields are everywhere in the universe, E-M theories of consciousness predict that everything, even a single electron, has some consciousness. The consciousness of a panpsychic universe is then like a fog of small particles of consciousness—leaving unsolved the problem of how these small particles are sometimes unified to give the clear and precise consciousness of space that we experience. So E-M theories fail to address the core nature of our own consciousness. But if consciousness arises from a rare state of matter such as a BEC, which exists in only one part of the brain, there is no difficulty from the integration and unity of our consciousness. We only require evolution to be able to discover and exploit some rare state of matter.

In these ways, the projective wave theory differs from electromagnetic theories of consciousness, and in my view is to be preferred over them. The most important reason is in the first point. The primary duty of any theory is to fit the data, and the projective wave theory agrees with the most important data about consciousness, which is the very good match between the geometry of spatial consciousness and the geometry of the real world.

This gives reason to predict a phenomenon that has not been directly observed in the brain—a wave excitation in the thalamus. There are well-known examples of effects that were theoretically predicted before they were directly detected. One example is Pauli’s prediction of the neutrino (Brown, 1978), which was not detected experimentally for another 25 years (Cowan et al., 1956). A more recent example, still not detected experimentally, is the prediction of dark matter in cosmology. The proposed thalamic wave is like the dark matter of the brain. Confirming or denying this dark matter can be a productive focus for experimental brain research.

Meanwhile, theoretical models of the brain, such as the Free Energy Principle and Active Inference (Friston et al., 2006; Friston, 2010; Parr and Pezzuolo, 2022), can be extended to include a central wave excitation. This is a possible new focus for theoretical brain research.

The projective wave theory is not just a theory of consciousness. It is a cognitive theory of how the brain works, and it differs radically from current pure neural theories. It is like a nuclear theory of the brain—seeing the brain not just as a complex neural computer, but as a neural computer sharing information with a central analogue model of 3-D space. The analogue model supports the vital function of spatial cognition, and is in effect the nucleus of the brain. If this nuclear model of the brain survives further development and testing, it will open avenues for new and exciting research.

## Data Availability

The data presented in the study are included in the article/supplementary material, further inquiries can be directed to the corresponding author/s.

## Footnotes

1 If a wave has wavelength *λ* and is travelling in a direction with unit vector **u**, then its wave vector **k** is given by **k** = 2π**u**/*λ*. Vectors in 3-space are denoted by bold letters.

2 Quantum mechanics is an exception to this; it violates classical locality, but in a way that is mathematically highly constrained, and is therefore testable.
